# Strategies of Classical Swine Fever Immune Evasion

**DOI:** 10.3390/ijms26167838

**Published:** 2025-08-14

**Authors:** Yuanji Zhang, Fangtao Li, Yebing Liu

**Affiliations:** National/WOAH Reference Laboratory for Classical Swine Fever, China Institute of Veterinary Drug Control, Beijing 100081, China

**Keywords:** CSFV, immune evasion, nonspecific immunity, adaptive immune response

## Abstract

Classical swine fever (CSF) is a highly contagious and lethal disease caused by classical swine fever virus (CSFV), and it is also a notifiable disease according to the World Organization for Animal Health. Owing to the continuous growth of the international trade in pigs and pig products, pig farming has become the pillar industry of the global livestock industry and is the most important source of animal protein for mankind. As a single-stranded RNA virus, CSFV can avoid being recognized and cleared by the host immune system through a variety of immune evasion strategies so that it persists in the host body and causes multisystemic pathology. CSF has also become one of the most serious infectious diseases affecting the pig industry, resulting in considerable economic losses to the pig industry. Therefore, understanding the main immune evasion mechanism of CSFV is very important for the prevention and control of CSF infection. This article reviews the main immune evasion mechanisms of CSFV, including the suppression of nonspecific immune responses; evasion of adaptive immune responses; and the regulation of host cell apoptosis and cell autophagy. CSFV affects type I interferon regulatory signals; the JAK-STAT signaling pathway; the RIG-I and NF-κB signaling pathways; immune cell function; the mitochondrial apoptosis pathway; and the endoplasmic reticulum stress apoptosis pathway; the PI3K-Akt signaling mediated AMPK-mTOR macroautophagy pathway through its structural proteins E^rns^ and E1 and E2; and the nonstructural proteins N^pro^, NS4B, and NS5A to achieve immune evasion. As our understanding of CSFV immune strategies continues to deepen, we believe that this understanding will provide new strategies for the development of new vaccines and novel diagnostic methods in the future.

## 1. Introduction

CSF is a highly acute and virulent infectious disease caused by classical swine fever virus CSFV. Pigs (including domestic pigs and wild boars) are the only susceptible hosts [1]. Since the first outbreak of CSF in Ohio in 1833 [2], it has caused considerable economic losses to the global pig industry and severely hindered its development. Therefore, it has been listed as a notifiable disease by the World Organization for Animal Health (WOAH) [3]. CSFV is a member of the *Pestivirus* genus of the *Flaviviridae* family. In addition, members of this genus also harbor bovine viral diarrhea virus-1 (BVDV-1), BVDV-2, and border disease virus (BDV) [4]. CSFV is an enveloped, icosahedral RNA virus with a 40–60 nm diameter, containing a 12.3 kb single-stranded positive-sense genome, contains a large open reading frame which encoding a 3898-amino-acid polyprotein cleaved into 4 structural proteins (C, E^rns^, E1, and E2) and 8 nonstructural proteins (N^pro^, P7, NS2, NS3, NS4A, NS4B, NS5A, and NS5B) [5]. Its single serotype includes three genotypes and eleven E2-based subtypes, with E^rns^ and E2 serving as key immunogens inducing neutralizing antibodies. CSFV primarily infects through the oronasal route, initially replicating in tonsillar epithelial cells before disseminating via lymphatic and circulatory systems to bone marrow, lymphoid organs, and macrophages within 48 h, preferentially targeting myeloid-derived cells like monocytes and alveolar macrophages [6].

CSFV is addicted to the lymphoid tissue of pigs and can cause damage to the immune system of pigs. After invading lymphocytes, CSFV can induce their apoptosis by mediating the expression of tumor necrosis factor-α (TNF-α), nuclear transcription factor κB (NF-κB), and interleukin (IL)-1α and IL-6, thus enabling immune evasion [7]. In the middle stage of infection, CSFV could cause T and B lymphocyte deletion, reducing T-cell proliferation and upregulating regulatory T cells (TREGs). In the late stages of infection, CSFV could induce host immune evasion by regulating apoptosis and autophagy and causing the loss of the porcine thymocyte-specific marker CD1 [7]. The pathogenesis of CSFV infection is a complex process, and immune evasion, as the main strategy of CSFV infection and proliferation, needs further study. This review outlines the current strategies by which CSFV achieves immune evasion, providing significance for the control of CSF and the development of new vaccines in the future.

## 2. Regulation of Innate Immune Response

The innate immune response, also known as innate immunity, is a normal physiological function which has an “inborn” nature. It serves as the first line of host defense for the animal body against the invasion of foreign pathogens and triggers the immune response, and then activates the processes of specific immunity. CSFV achieves its immune suppressive effects primarily by inhibiting various pathways of innate immune molecules, such as complements, cytokines, and enzymatic components [1], as well as targeting innate immune cells, including phagocytes, NK cells, and dendritic cells. This multifaceted mechanism facilitates immune evasion and confers a survival advantage to the virus during the early phase of infection [8].

### 2.1. Suppressing Interferons (IFNs)

Upon viral infection, cells produce type I IFNs, which are secreted extracellularly and bind to specific receptors on the surface of target cells. This binding activates the JAK-STAT signaling pathway, inducing the transcription of interferon-stimulated genes that lead to the production of a range of antiviral proteins. CSFV suppresses type I IFNs synthesis through dual mechanisms targeting critical immune pathways. The nonstructural protein N^pro^ binds interferon regulatory factor 3 (IRF3), triggering its ubiquitin-proteasome degradation to block IFN-β transcription [9,10]. Concurrently, the structural protein Erns degrades viral double-stranded RNA (dsRNA) via RNase activity, impairing RIG-I/MDA5 recognition and the MAVS-dependent signaling required for IFN induction [11].

Even if host cells successfully synthesize interferons, CSFV can still evade the immune response by blocking the transmission of interferon signals. E^rns^ suppresses Toll-like receptor (TLR) 7-dependent IFN-α induction in plasmacytoid dendritic cells (pDCs) after infection [12]. Previous studies have found that there is an increase in the expression of nuclear factor κB inhibitor α (NF-κBIA) in peripheral blood leukocytes [13], which may be induced by the N^pro^ protein under the mediation of the tumor necrosis factor α (TNF-α) [14]. Although N^pro^ and E^rns^ can both inhibit the production of type I IFNs or block interferon signaling, the N^pro^ primarily suppresses extracellular and nuclear signals, while the E^rns^ specifically inhibits the signaling recognition of intracellular dsRNA. NS5A can inhibit the activation of the JAK-STAT signaling pathway by inhibiting STAT1 phosphorylation to diminish the cascade of immune responses mediated by the antiviral gene interferon-stimulated gene 15 (ISG15) [12,15]. In addition, NS5A may affect NF-κB activity by inhibiting NF-κB nuclear translocations or promoting the production of inhibitory kappa B kinase (IKK) complexes [15,16]. However, the regulatory mechanism of NF-κB and the inhibition mechanism of TLR7 remain to be verified experimentally.

### 2.2. Blocking the Signalling Pathways Affected by PRRs

The innate immune system, through the pattern recognition receptors (PRRs), identifies PAMPs or DAMPs to detect pathogens or damage. When PRRs are activated, it will activate gene transcription, leading to the release of cytokines, interferons, and other signaling molecules [17]. Current research indicates that the main PRRs associated with swine fever include TLR 3, RIG-I-like receptors (RLRs), and MDA 5 [17,18,19]. After CSFV infection in pigs, the transcriptional levels of TLR3, TLR5, and TLR9 in macrophages are significantly reduced [8]. Another experiment demonstrated that the mRNA and protein levels of TLR2, TLR4, and TLR7 are increased in response to CSFV infection, whereas TLR3 is decreased [20]. NS4B was also found to inhibit the TLR3 signaling pathway, thereby suppressing IRF3 protein translation and NF-κB p65 phosphorylation [20].

RIG-I and MDA5 are cytoplasmic receptors essential for detecting viral RNA. RIG-I initiates antiviral responses by sensing exogenous RNA, while MDA5 specifically recognizes viral dsRNA [19]. Previous studies have shown that the overexpression of Hemoglobin (HB) can inhibit the replication and proliferation of CSFV. When the RIG-I pathway is blocked, HB cannot inhibit the proliferation of CSFV [21]. Furthermore, RIG-I gene knockout experiments have confirmed that RIG-I is an important sensor for recognizing CSFV infection [19]. Studies have shown that primary infection with CSFV leads to the high expression of MDA5 [19], while cells with low MDA5 knockdown exhibit little production of IFN or inflammatory cytokines after infection with CSFV [16,22]. Recent experiments have found that IFNs may act together with MDA5 to suppress CSFV replication [23,24].

### 2.3. Regulating Interleukin (IL)

ILs play crucial roles in the innate immune response; they provide immune protection by regulating inflammatory reactions and mediating the activation, proliferation, and differentiation of T and B cells. CSFV infection primarily affects the production of IL-1β, IL-6, IL-10, and IL-12 to suppress the host immune response and achieve immune evasion. After peripheral blood macrophages are infected with highly pathogenic CSFV, the expression of the genes encoding IL-1α, IL-1β, IL-6, and IL-12 p35 is significantly increased [7,25].

In vitro experiments have shown that infection by CSFV leads to an increase in IL-1α expression [26]. Its viral pore protein p7 can induce the secretion of IL-1β by macrophages, promote inflammatory response and enhancing the replication of CSFV [27,28]. However, recent findings suggest that tryptophan metabolism mediated by indoleamine 2,3-dioxygenase 1 (IDO1) negatively regulates the NF-κB signaling pathway, reducing the expression of IFN-α, IFN-β, and IL-6 [29]. The NS4A and NS4B proteins interact with MAVS and promote the expression of IL-8 by blocking the activation of IRF3 and NF-κB [30,31]. The dendrites in the spleen can regulate the expression of IL-10 during the later stages of CSF infection [32]. Vaccination experiments also found that IL-10 and IL-12 may be related to the pathogenicity of CSFV [33]. Other vaccination experiments have confirmed that IL-18 plays a key role in defending against CSFV infection; the coadministration of IL-12 also increases the secretion of neutralizing antibodies [34].

## 3. Regulation of Adaptive Immune Response

Adaptive immune responses involve a complex network of effector cells and are divided into two main parts: humoral immunity and cell-mediated immunity. It is generally believed that the humoral immune response leads to the clearance of viruses in the circulatory system and prevents the spread of the virus within the host, whereas the cell-mediated immune response clears infected cells [35]. After activation, both cellular and humoral immune responses can work in concert to control viral infections. However, if one aspect is insufficient, the antiviral effect may be compromised. CSFV primarily replicates by inhibiting antigen presentation, disrupting T-cell-mediated immune responses, and suppressing B-cell activation and antibody production.

### 3.1. Suppressing Antigen Presentation

Dendritic cells (DCs) are professional antigen-presenting cells with potent antigen-presenting and immune-regulatory capabilities. After DCs capture pathogens and present antigens, this will induce the differentiation of T lymphocytes, or directly activate B lymphocytes [35]. CSFV can infect DCs of monocytic and bone marrow origin and replicate effectively within them; however, the CSFV infection of DCs does not induce a strong immune response.

Research has demonstrated that the NS3 protein can mediate ubiquitination degradation of monoglobin to inhibit the expression of MHC-I antigen presentation-related proteins [36]. Additionally, a fifteen-amino acid peptide of CSFV NS2-3 can promote the T-cell expression of MHC-II and MHC-I by stimulating the secretion of IFN-γ [37]. Studies involving the coadministration of IFN-γ with a CSFV attenuated strain vaccine have revealed that MHC-I and MHC-II expression is upregulated in pigs immunized with IFN-γ [38]. CSFV infection does not activate the expression of MHC-I, MHC-II, and CD80/86 molecules [32]. When infected DCs were stimulated with TNF-α/IFN-α or poly (I:C), the expression of MHC-I, MHC-II, or CD80/CD86 molecules increased [39]. This suggests that infection with CSFV inhibits DC activation and decreases the recognition of relevant MHC-I and MHC-II molecules, thereby inhibiting antigen-presentation pathways and weakening T-cell-mediated immune responses.

### 3.2. Suppressing T-Cell Immune Responses

T cells are crucial components of the adaptive immune system. According to the different CD molecules, T cells can be divided into CD4+ T cells and CD8+ T cells [40]. To initiate the activation of effective cytotoxic T lymphocytes (CTLs) and stimulate antibody production in B cells, CD4+ T cells primarily produce multiple cytokines [41]. CD8^+^ T cells are mainly responsible for mediating cellular immune responses [42]. Infection with a highly virulent strain of CSFV can lead to a rapid decrease in the number of peripheral blood CD8+ lymphocytes, and a similar trend is observed in CD4+ T lymphocytes [42]. Piriou L. et al. reported that in the first week after CSFV infection, there was no significant change in the distribution of T lymphocyte subsets; as the terminal phase approaches death, there is a significant decrease in the CD4-CD8- T lymphocyte subset [43]. Additionally, vaccination experiments have indicated an increase in the CD8+CD25+ lymphocyte subset, which is similar to cytotoxic T cells, leading to reinforced immunity to the vaccine [44].

During the early stages of CSFV infection in DCs, the transient expression of IL-12, IL-18, and IFN-γ indicates the onset of humoral immunity; however, immune cells are impaired at later stages due to high levels of TNF-α and the deficiency of IFN-α and IL-10 [32]. In vitro studies have found that the frequency of CSFV-specific IFN-γ+ CD8+ T cells is inversely correlated with the viral load in individual animals, but the specific reasons remain to be elucidated [45]. Another study found two novel T-cell epitopes on the E2 protein, which are related to the specific regulation of IFN-γ secretion by CD8- T cells in response to CSFV [46]. These studies show that the reduction in IFN-γ levels reflects a decrease in the intensity of T-cell responses, causing persistent infections [38,47]. Moreover, CSFV can upregulate the inhibitory cytokines IL-10 and TGF-β1, inhibiting the antigen-presenting function of DCs [33,48].

### 3.3. Suppressing B-Cell Immune Responses

B cells play an essential role in viral infections, producing specific antibodies to clear the virus and prevent it from invading host cells. Additionally, B cells play a significant role in the formation of immune memory, providing long-term immune protection for the body. After CSFV infection, not only is the T-cell immune response suppressed, but the B-cell immune response is also significantly affected, impacting antibody production and the establishment of immune memory [49].

After pigs are infected with CSFV, the number of B lymphocytes in the peripheral blood sharply decreases. The decline in B lymphocytes caused by the low-virulence strain occurred 3 days postinfection, whereas a sharp decrease in B lymphocyte numbers was observed on the second day after inoculation with the high-virulence strain [50]. Regardless of whether the virulence of the strain or the dose of inoculation is the same, once pigs are infected with CSFV, the total number of B lymphocytes will eventually be reduced to a similarly low level, which is consistent with the inability to produce neutralizing antibodies[43]. CSFV infection can also damage the germinal centers of lymphoid tissue, hindering the maturation of B lymphocytes and leading to a depletion of B lymphocytes in the circulatory system and lymphoid tissues, the atrophying of the thymus and leukopenia, and destruction of the bone marrow [32].

## 4. Regulation of Apoptosis

Apoptosis is important for a host to resist virus invasion; it can quickly destroy the infected cell and prevent the replication and spread of the virus [51]. Apoptosis can also promote T lymphocyte activation and enhance the phagocytic function of antigen-presenting cells, accelerating the immune system’s clearance of viruses [51]. CSFV can directly or indirectly regulate the apoptosis pathway, inhibit the apoptosis of infected cells, and promote the continuous replication of the virus through a double-inhibition effect, while the selective induction of the apoptosis of immune cells by the virus weakens the antiviral defense, thus providing conditions for the replication and transmission of the virus and achieving immune evasion.

### 4.1. CSFV Infection Induces Apoptosis in Target Cells

CSFV contains dsRNA that can induce apoptosis, but this effect can be inhibited by the Npro protein synthesized by the virus [52]. Bensaude et al. reported that infection of cells by CSFV leads to increased NF-κB activity, then regulates cytokine expression, thereby inhibiting the apoptosis induced by the virus [53]. Ruggli et al. reported that the N^pro^ protein of CSFV can inhibit the synthesis of IFN-α/β induced by polyI:C after infection with macrophages, thereby inhibiting apoptosis [52]. Glyceraldehyde-3-phosphate dehydrogenase (GAPDH), a key glycolytic enzyme, serves as a universal mediator of apoptosis, and its expression level in apoptotic cells is three times greater than that in nonapoptotic cells [54]. Proteomic analysis of PK-15 cells infected with CSFV revealed that GAPDH expression was significantly downregulated and that CSFV-infected PK-15 cells did not exhibit cytopathic effects or induce apoptosis [55]. Conversely, CSFV infection induces an 8-fold increase in GAPDH expression in the peripheral blood mononuclear cells (PBMCs) of the host [56].

Additionally, in vitro experiments have indeed shown that CSFV infection can induce apoptosis in various immune cells. Summerfield et al. reported that, after CSFV infection, both high and low virulence results in the massive apoptosis of leukocytes early and late in the infection [57]. Sanchez-Cordon et al. confirmed that, after CSFV infection in pigs, a large amount of apoptosis occurred in both lymphocytes and macrophages, which was related to the increased expression of TNF-α, IL-1α, and IL-6 induced by CSFV [7]. Meanwhile, the high expression of NF-κBIA following CSFV infection also inhibits the action of NF-κB, promoting cell apoptosis [13].

### 4.2. CSFV Indirectly Induces Apoptosis

In recent years, a growing body of evidence has suggested that the phenomenon of lymphocyte depletion is due to apoptosis, which is related mainly to apoptotic factors such as TNF-α and IL-1α [58]. The main mechanisms of B cell depletion caused by CSFV infection are as follows: the virus induces the cytokines to disrupt the balance of the normal cellular Fas-FasL (Fas/Fas ligand) signaling pathway in these immune cells, triggering apoptosis [58]. Moreover, most infected cells do not undergo apoptosis; instead, uninfected cells mainly undergo apoptosis [59].

Further research revealed that CSFV is significantly associated with the activation of caspase-3 and caspase-9 and that cytotoxic cytokines and reactive oxygen species do not play major roles in cell apoptosis [60]. After CSFV infection, genes related to caspases 3 and 7 are upregulated in the host; whereas, the expression of caspase-6 is downregulated [61]. In the late stages of infection, the Cyt-C pathway associated with ROS in cell apoptosis is inhibited [62,63], and there is no significant change in the expression of the key caspase-12 gene in the endoplasmic reticulum stress pathway [60].

Additionally, the host alters the expression of several antioxidant stress protein genes to resist virus-induced apoptosis, such as HO-1, Hsp27, PGAM1, and Prs-1. The upregulation of HO-1 and Hsp27 genes in CSFV-infected cells inhibits apoptosis induced by oxidative stress and may trigger an antiapoptotic response [62,64]. The upregulation of PGAM1 may promote cell apoptosis and facilitate persistent infection [62], while the downregulation of Prx-1 may imply the promotion of cell apoptosis and a reduction in the antiviral activity of CD8+ T cells [62], but the specific mechanisms of its action still need to be elucidated. Additionally, the specific roles of other apoptosis-related genes, such as cytochrome P450, caspase-1, p75 apoptosis, death domain, TCTP, MAPKAPK3, and PRL6 are altered [65]. The specific roles of these genes in virus-regulated apoptosis remain to be further elucidated.

## 5. Regulation of Autophagy

Autophagy is a cellular degradation and recycling process, it is essential for cells to maintain homeostasis, renew damaged organelles, and clear invading pathogens [66]. The key regulators of autophagy include the ATG1/ULK1 and PI3K protein kinase complexes [67], with mTORC1 acting as a critical switch in autophagy when inhibited under stress conditions such as starvation or intracellular pathogen infection, activating autophagy [68]. Some viruses have evolved strategies to either inhibit autophagy or hijack autophagosomes for replication. CSFV infection increases autophagy-like vesicles and triggers complete autophagy, as evidenced by the conversion of LC3-I to LC3-II and the binding of ATG12 with ATG5 [69]. In CSFV cell infection experiments, the high expression of autophagy markers ATG5 and BECN1 (Beclin 1), along with the degradation of SQSTM1 (Sequestosome 1), indicates that CSFV infection triggers a complete autophagy response. In-depth research reveals CSFV E2 and NS5A proteins can bind to the autophagy-related factors LC3 and CD63 to boost viral replication [69]. Later, it was found by Pei’s that CSFV infection raises mitochondrial DNA copies and ROS production, and enhances the RLR signaling linked to apoptosis [70]. This suggests that CSFV can delay cell apoptosis by downregulating ROS-dependent RLR signaling, leading to persistent viral infection. CSFV affects the PINK1/Parkin pathway by inducing mitochondrial division and autophagy through Drp1 translocation and the upregulation of Parkin and PINK1, thereby achieving persistent infection [71].

CSFV can also utilize the mTORC1 pathway for viral replication and immune evasion [72]. Host restriction factor serine incorporator 5 (SERINC5) inhibits CSFV-induced autophagy through the MAPK1/3-mTOR and AKT-mTOR pathways, promoting apoptosis [73]. CSFV can also induce ER stress through the PERK and IRE1 pathways to mediate complete autophagy [74]. Additional research indicates that CSF-mediated autophagy is driven by the activation of MTOR through [Ca^2+^]cyto-CAMKK2-PRKAA, the activation of MAPK1/3, and the inhibition of AKT [75]. Moreover, CSFV can propagate through extracellular vesicles (EVs) derived from macroautophagy/autophagy, revealing a new immune evasion mechanism [76]. Fan et al. reported that CSFV infection suppresses autophagy receptor nuclear dot protein 52 kDa (NDP52) expression, reducing CSFV entry into autophagosomes [77].

Studies have shown that the NS3, C, NS5A, and NS4A proteins of CSFV are involved in autophagy regulation. The NS3 protein can inhibit the expression of lactate dehydrogenase B (LDHB), promote mitochondrial autophagy, and then inhibit the activation of NF-κB signaling pathways, but the underlying mechanism is still unclear [78]. The C protein can degrade OGDH and regulate the AMPK-mTOR pathway to modulate the IRF3-IFN-β network and then promote CSFV replication [79]. Silencing HK2 can induce autophagy, reducing the interferon and NF-κB signaling pathways so that they inhibit CSFV infection-induced cell apoptosis [80]. The NS5A and NS4A proteins activate the AMPK-mTOR signaling pathway by upregulating the expression of Pyruvate kinase M2 (PKM2), inducing mitochondrial autophagy [81]. NS5A can also increase LC3 levels and increase the expression of Parkin and PINK1, then promote CSFV autophagy [82,83]. Further studies also found that NS5A can also regulate autophagy through ROS expression levels [84]. The NS4A protein can promote the expression of TRIM25, and target the autophagy degradation of RIPK3 to block necrosis, but whether it is the main mediator of autophagy remains to be determined [85,86]. These findings highlight the multifaceted strategies of CSFV to modulate autophagy for immune evasion and efficient replication.

## 6. Conclusions and Perspectives

**Figure 1:**
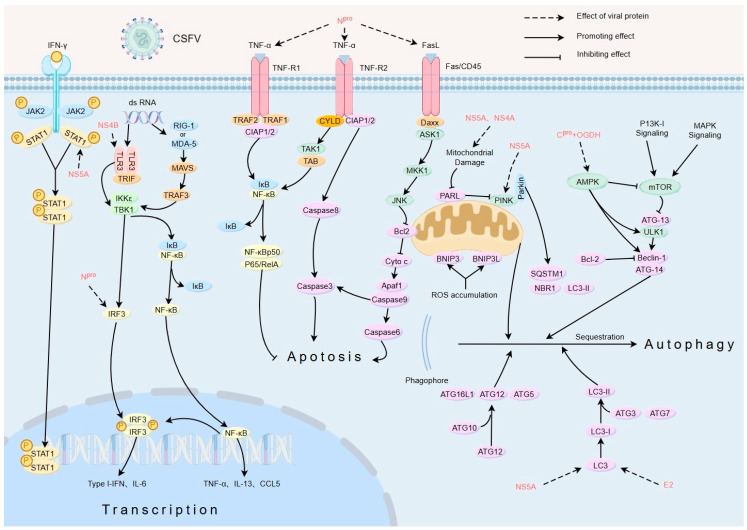
CSFV viral proteins act on different signaling pathway proteins (the red font indicates the viral proteins).

A comprehensive review of the strategies used by CSFV to evade the immune system reveals a complex and multifaceted approach that emphasizes the adaptability and persistence of the virus within its host. Understanding the immune evasion mechanism of CSFV is very important for its pathogenesis and is highly important for the control and prevention of CSF in the global pig industry. Figure 1 summarizes the immune evasion process of several key CSFV proteins acting on host cells.

The ability of CSFV to suppress innate and adaptive immune responses is a key factor in its virulence. By interfering with interferon production and signaling, blocking the pattern recognition receptor (PRR) signaling pathway, and regulating the expression of interleukins and coagulation factors, CSFV establishes persistent infection and causes significant immunosuppression [19,25]. In addition, CSFV’s manipulation of the apoptosis and autophagy pathways represents a complex strategy by which viruses evade host immune defense after adapting to host immunity [87]. By inducing the apoptosis of immune cells and inhibiting the apoptosis of target cells, CSFV not only disables the host’s antiviral response but also creates a favorable environment for viral replication. Similarly, the way in which CSFV uses the autophagy pathway to replicate and spread itself highlights the complex relationship between CSFV and its host [81].

A deeper understanding of these immune evasion strategies will be critical for the development of novel, highly effective approaches for vaccine protection and diagnostics. For example, an African swine fever (ASFV) recombinant strain containing the E2 protein of CSFV showed complete protection against ASFV and CSFV [88], which provides insights into the development of bivalent vaccines against ASFV and CSFV. The constructed E2 protein inserted into porcine *Circovirus* type 2 (PCV2) and Pseudorabies virus (PRV) also showed good immune effects [89,90], providing a direction for the development of multivalent vaccine in the future. The monoclonal antibodies (mAbs) against the E2 and E^rns^ fusion proteins could be used to distinguish the vaccine strains from the field isolates effectively, which provides an idea for the diagnosis of the fusion antigen [91]. In addition, the exploration of host-related protein factors and cellular pathways affected by CSFV provides theoretical support for the discovery of new antiviral strategies. For example, the IFN-γ-inserted E2 fusion protein elicited stronger immune responses and improved the immunogenicity of the CSFV E2 subunit vaccine [92]. By adding drugs that restore the function of antigen-presenting cells or promote IFN-γ [38] secrection, the immunogenicity, protection, and duration of current vaccines are significantly improved.

With the increasing popularity of mRNA vaccine research, the research on the immune efficacy and detection methods of CSFV and nanoparticle complexes has become an important research direction. The dual antigen binding of the E2 protein and the PCV2 Cap protein to nanoparticles (NPs) can enhance specific IgG responses, improve antigen recognition and presentation ability, and enhance neutralizing antibody levels [93,94]. Recently, it was found that the delivery of the E2 dimer by NPs alone enhanced Th2 responses and improved Th1 responses, and the activated immune cells significantly increased the immune response [95]. Furthermore, current studies on the interaction of CSFV with various cell death pathways, such as the autophagy, apoptosis, and pyroptosis pathways, provide new paths for the development of targeted therapies and new targets for drug delivery.
